# Remote targeted electrical stimulation

**DOI:** 10.1088/1741-2552/acd95c

**Published:** 2023-06-09

**Authors:** Taylor Webb, Rahul Cheeniyil, Matthew Wilson, Jan Kubanek

**Affiliations:** 1 University of Utah, 36 S Wasatch Dr, Salt Lake City, UT, 84112, United States of America

**Keywords:** incisionless, noninvasive, induction, Lorentz force, ultrasound, magnetic field, neuromodulation

## Abstract

*Objective:* The ability to generate electric fields in specific targets remotely would transform manipulations of processes that rest on electrical signaling. *Approach:* This article shows that focal electric fields are generated from distance by combining two orthogonal, remotely applied energies—magnetic and focused ultrasonic fields. The effect derives from the Lorentz force equation applied to magnetic and ultrasonic fields. *Main results:* We elicited this effect using standard hardware and confirmed that the generated electric fields align with the Lorentz equation. The effect significantly and safely modulated human peripheral nerves and deep brain regions of non-human primates. *Significance:* This approach opens a new set of applications in which electric fields are generated at high spatiotemporal resolution within intact biological tissues or materials, thus circumventing the limitations of traditional electrode-based procedures.

## 1. Introduction

Many processes in biology and nature rest on electrical signal transduction. Bioelectric medicine and neuromodulation aim to manipulate these signals in a targeted manner with the goal to restore or augment function (van Balken *et al* 2004, Larson 2014, Lempka and Patil 2018, Peeples 2019). Currently, circumscribed targeting of electric fields is achieved by inserting or implanting electrodes into the intended locations (van Balken *et al* 2004, Larson 2014, Lempka and Patil 2018). This requires invasive steps that limit the flexibility and safety of this traditional approach (Bergey *et al* 2015, Tonge *et al* 2015, Sinai *et al* 2019, Giordano *et al* 2020).

Noninvasive approaches, which have rested on electrical, magnetic, and electromagnetic fields, have much greater flexibility in that they do not incur additional risk to subjects when modulating multiple sites. However, these approaches do not have the necessary spatial resolution to modulate specific neural circuits at depth. For instance, electric fields generated with current noninvasive approaches, including electroconvulsive therapy (ECT) or transcranial direct or alternating current stimulation are relatively broad (Lisanby 2007, Caumo *et al* 2012, Herrmann *et al* 2013). Consequently, the sizable activation of the brain associated with ECT often results in cognitive side effects (Ingram *et al* 2008). The spatial resolution of these methods can be improved using multi-channel approaches (Shin *et al* 2018, Vargas *et al* 2020, Pena *et al* 2021) or spatially interfering fields (Nemec 1959, Grossman *et al* 2017), but the resulting fields are still broad with respect to the dimensions of neural circuits. Transcranial magnetic stimulation (TMS) uses pulses of magnetic fields to noninvasively induce electric fields in the brain. TMS can produce appreciable effects in the cortex and ameliorate symptoms of depression (George *et al* 2000), but the approach cannot directly and focally modulate deep brain regions.

Electromagnetic waves currently cannot be used to modulate deep brain targets in a focal manner. At high frequencies (light or infrared), electromagnetic waves are severely attenuated by the skull or superficial tissue layers (McCormick *et al* 1992). At lower frequencies, the waves (microwaves) can penetrate into depth, but microwaves at the relevant neuromodulatory doses damage mitochondria and possibly other cellular structures (McRee and Wachtel 1980, Hao *et al* 2015). At yet lower frequencies (radio range), the wavelength is too broad—dozens of centimeters or meters—to allow for focal stimulation (Lustenberger *et al* 2013).

Ultrasonic waves combine depth penetration and safe application. Ultrasound can effectively modulate excitable cells at high frequencies—above 10 MHz—at which there are strong radiation forces that mechanically displace membranes and activate ion channels (Menz *et al* 2013, Kubanek *et al* 2016, 2018, Prieto *et al* 2018). However, ultrasound at such high frequencies is severely attenuated by the human skull (Fry 1977, Fry and Barger 1978); for this reason, frequencies below 1 MHz have been used for transcranial therapies (Kubanek 2018). Ultrasound can modulate excitable structures also at lower frequencies (Naor *et al* 2016, Blackmore *et al* 2019), but strong effects that are based on established biophysical principles, remain elusive.

**Figure 1.**
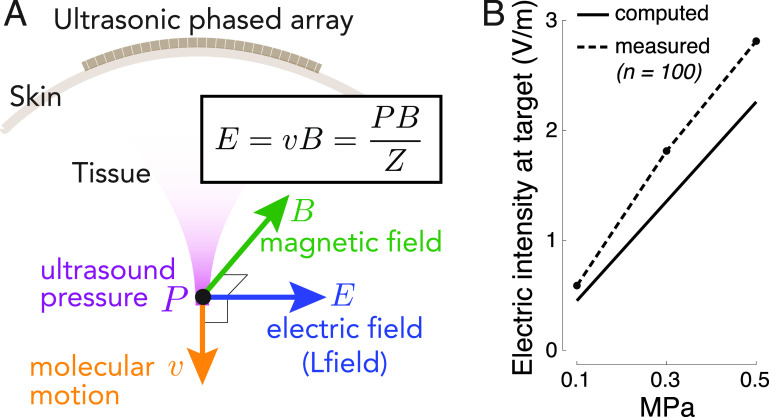
Remote generation of focal electrical fields. (A) Concept. An ultrasonic transducer array programmatically focuses ultrasound into a target of interest. An ultrasound wave, focused into a target with acoustic impedance *Z*, induces in the target motions of molecules with velocity $v = \frac{P}{Z}$. The pressure *P* (and so the velocity *v*) are maximal at the target. When the wave is emitted in a direction perpendicular to magnetic field *B*, so that the velocity vector is perpendicular to *B*, the target experiences localized electric field $E = \frac{P B}{Z}$. (B) Validation. Electric field at target measured with a pair of electrodes inside a 7 T field when a 258 kHz focused ultrasound of the pressure amplitude indicated on the abscissa is delivered into the target (figure 2(A) and methods). The measurements align with the theoretical values computed from the Lorentz equation for this magnetic field strength (*n* = 100 measurements, mean ± s.e.m.; the error bars are smaller than the symbols).

Recent advances in the production of strong magnetic fields (Battesti *et al* 2018) make it possible to noninvasively generate localized electric fields. This can be achieved via combined use of focused ultrasound and a magnetic field. Specifically, when a charged molecule *q* moves at a velocity $\vec{v}$ in magnetic field $\vec{B}$, the molecule experiences the Lorentz force $\vec{F} = q(\vec{v} \times \vec{B})$ with intensity $\vec{E} = \frac{\vec{F}}{q} = \vec{v} \times \vec{B}$. To produce localized electric field, the molecular motion $\vec{v}$ should occur only in the target of interest. Critically, this targeting can be achieved using focused ultrasonic waves. Ultrasound—a mechanical pressure wave—displaces molecules at its target with velocity $v = \frac{P}{Z}$ (Cobbold 2006), where *Z* is a constant of the medium, ‘acoustic impedance’. Thus, acoustic waves delivered into a target perpendicularly to a magnetic field produce in the target electric field intensity $E = \frac{PB}{Z}$. This intensity points in the direction that is perpendicular to both constituents (figure 1(A)). As a consequence of this electric field, positively and negatively charged molecules are pulled in opposite directions, inducing electric currents. Sound waves alone would displace positively and negatively charged molecules in the same direction, thus no gradient of charge and so no electric field would be created; the magnetic field is a critical addition. The temporal profile of the evoked field *E*(*t*) corresponds to $E(t) = \frac{P(t)B(t)}{Z}$. Therefore, the induced waveform can be controlled by the temporal profile of the ultrasonic, magnetic, or both, fields.

We refer to the resulting electric field as Lfield and the resulting stimulatory effects as Lstim, given their origin in the Lorentz equation and their electrical and local nature. While theoretically plausible (Edrich and Zhang 1993, Norton 2003, Kishawi and Norton 2010, Yuan *et al* 2016), whether the effect is applicable to manipulations of biological or physical systems has been unclear (Wang *et al* 2019).

## 2. Methods

### 2.1. Ultrasonic apparatus

**Figure 2.**
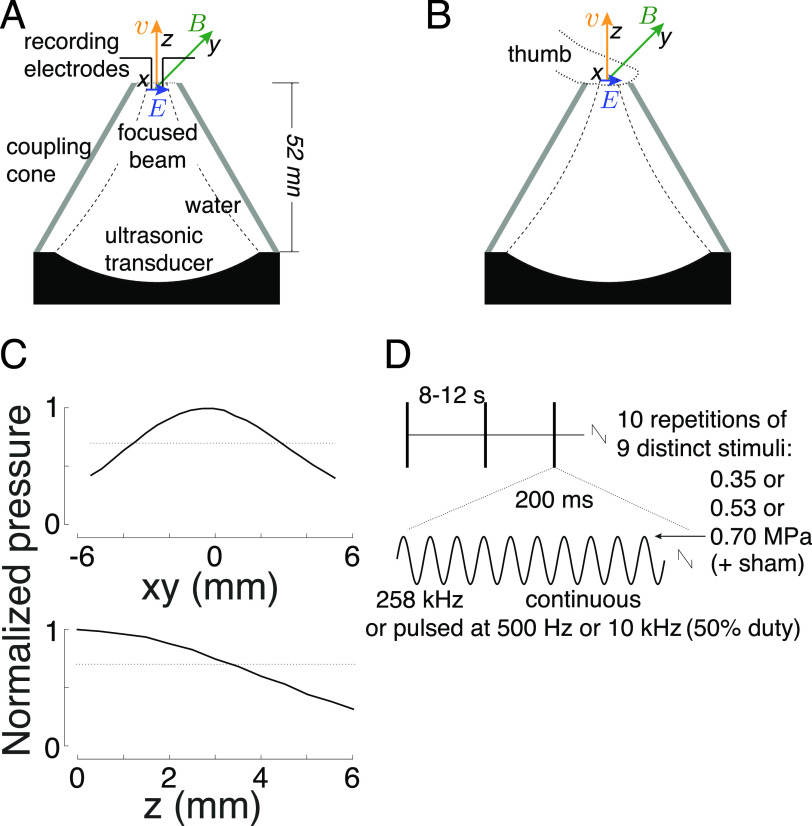
Apparatus and stimuli. (A) Apparatus used for recordings. A focused ultrasound transducer delivered a 258 kHz stimulus into a target inside a 7 T scanner. Two electrodes positioned into the target measured the induced electric field. The measurements were performed in the indicated geometry and following a rotation of the setup 90^∘^ with respect to the magnetic field. The coupling cone was filled with saline. The inter-electrode distance was 3 mm. (B) Apparatus used for nerve stimulation. A focused ultrasound transducer delivered a 258 kHz stimulus into a subject’s thumb using a coupling cone filled with degassed water. The stimulation was performed inside a 7 T scanner or 3 m away from it. Subjects were instructed to place the thumb so that it pointed perpendicularly to the magnetic and ultrasonic fields. (C) Peak-normalized ultrasound pressure field. The pressure profile was averaged over the x and y dimensions. The dotted lines show the 0.707 (0.5) pressure (intensity) levels to characterize the fields using full-width-at-half-maximum values. The full-width-at-half-maximum (FWHM) diameter was 6.5 mm in the xy-dimension, and focal length (z-dimension) 3.3 mm. (D) Peripheral nerve stimulation parameters. Each subject experienced ten repetitions of ten distinct stimuli, including sham. The stimuli, 200 ms in duration, were selected randomly and delivered every 8–12 s. We tested three pressure levels and continuous and pulsed (500 Hz, 10 kHz frequency, 50% duty) stimuli.

The ultrasonic stimuli for the field assessment and the peripheral nerve stimulation were generated using a focused, MRI-compatible ultrasonic transducer (H-115, Sonic Concepts, 64 mm diameter, 52 mm focal depth). The transducer was operated at 258 kHz. A water-filled coupling cone (1 mm-thick plastic) was used to focus the ultrasound into the target (figure 2). The height of the cone was 52 mm and its diameter 70 mm. The cone’s aperture had a 16 mm diameter at the ultrasound target. Stimuli were generated by a custom Matlab program that produced the stimulation waveforms in a programmable function generator (33520B, Keysight). The signals were amplified using a 55 dB, 250 kHz–30 MHz power amplifier (A150, Electronics & Innovation).

### 2.2. Measurements of the ultrasonic fields

The pressure fields were measured in free field—in a water tank—at the location of the ultrasound target. The pressures were measured using a capsule hydrophone (HGL-0200, Onda). The hydrophone was calibrated between 250 kHz and 40 MHz and secured to 3-degree-of-freedom programmable translation system (Aims III, Onda). The spatial distribution of the generated ultrasound pressures is shown in figure 2(C). Notably, the hydrophone measurements incur about 1 dB error (HGL-0200, Onda). This can introduce a discrepancy between the theoretical and measured fields (figure 1(B)).

### 2.3. Magnetic field

The measurements and peripheral nerve stimulation was performed inside a 7 T MRI scanner (Bruker BioSpec). The transducer was positioned inside the bore at a distance 20 cm from the exit plane of the bore. The static magnetic field inside the bore is considered relatively uniform. The magnetic field pointed in direction perpendicular to the ultrasonic field (figure 2).

### 2.4. Measurements of the generated electric fields

The generated electric fields were measured using a pair of copper electrodes positioned at the ultrasound target (figure 2(A)). The inter-electrode distance was 3 mm. The coupling cone was filled with saline. The electrodes were insulated with only their tip exposed to the medium. We collected 100 repetitions of 50 ms continuous, 258 kHz tone burst at pressures amplitudes of 0.1, 0.3, and 0.5 MPa. For each repetition, we measured the peak amplitude of the voltage elicited between the electrodes, and averaged the 100 values for each pressure together. We made sure the measurements were not influenced by potential artifacts associated with the ultrasound. To do so, we contrasted the effects of the default geometry Lfield (figure 2(A)), with that rotated 90^∘^, and subtracted the respective voltage amplitudes.

### 2.5. Computation of the generated electric fields

The generated electric field obey the Lorentz equation, $E = \frac{P B}{Z}$. In this equation, we used the acoustic impedance of *Z* = 1.58 MRayl (Riis and Kubanek 2021). For the measurements to be deterministic, the electrodes were inserted into the saline at a depth of 1.5 mm. This corresponds to one-quarter wavelength. We took this step so that the electrode tips were positioned at a defined location within the antinode of the wave reflected from the water-air interface. The antinode experiences double the pressure, thus leading to $E = \frac{2 P B}{Z}$ (figure 1(B)).

### 2.6. Nerve stimulation

The study was approved by the Institutional Review Board of the University of Utah. Eighteen subjects (6 females, 12 males, aged between 21–38 years) participated in the study. All subjects provided an informed consent. Subjects were asked to gently rest the thumb of their right hand on a plastic coupling cone filled with degassed water (figure 2(B)). Subjects had their eyes closed and wore noise-cancelling earmuffs (X4A, 3 M; 27 dB noise reduction) so that they could fully focus on the stimuli. Subjects could not hear or see the stimuli or their generation.

### 2.7. Stimuli

The stimulation was performed inside the bore of the 7 T MRI scanner or at a 3 m distance away from it. The stimulation order was randomized, without replacement, such that half of the subjects experienced the stimulation in the scanner first and the other half outside of the scanner first. Subjects were asked to place the finger on the aperture in the direction perpendicular to the ultrasonic and magnetic fields (figure 2(B)) to maximize the Lstim effects.

We used nine distinct stimuli, of three pressure levels and three distinct waveforms (figure 2(D)). A tenth, sham stimulus, delivered negligible pressure (5 kPa, corresponding to the noise level of the amplifier-transducer output) under the same conditions. The parameters were chosen to provide safe and effective stimulation. The transducer’s fundamental carrier frequency was 258 kHz. The duration of each stimulus (200 ms) was chosen to provide ample time for potential integrative effects. The peak pressure amplitudes of the ultrasound measured at the center of the aperture were 0.35 MPa, 0.53 MPa, and 0.7 MPa. The peak pressures were chosen such as to trigger appreciable electric intensities at target (up to 3.1 V m^−1^), but low enough to comply with the $I_\textrm{SPPA}$ Track 3 510(k) recommendation for each pulse and within the $I_\textrm{SPTA}$ recommendation over the course of the experiment (see Stimulus safety), and low enough to prevent unpleasant nociceptive responses. The stimuli were either continuous (200 ms of tone burst) or pulsed at 500 Hz or 10 kHz, both at 50% duty. We added the pulsed stimuli under the hypothesis that pulsed stimuli may provide multiple onset responses (Grossman *et al* 2017), thus amplifying the stimulation. The effect of Lstim was observed regardless of whether the stimulus was continuous or pulsed; there was only a weak (table 1) interaction of the stimulus waveform and magnetic field.

**Table 1.** Summary of the effects. The effects of magnetic field (*M*), ultrasound pressure (*P*), and stimulus waveform (*W*; continuous or pulsed) on the frequency of nociceptive (left column) and tactile responses (right column). These effects were assessed using a three-way ANOVA that featured the three main effects and all possible interactions. Bold entries are significant (*p* < 0.05).

	Nociceptive	Tactile
*M*	$\boldsymbol{\lt0.001}$	**0.0042**
*P*	$\boldsymbol{\lt0.001}$	$\boldsymbol{\lt0.001}$
*W*	$\boldsymbol{\lt0.001}$	$\boldsymbol{\lt0.001}$
*M* × *P*	**0.0012**	0.23
*M* × *W*	**0.043**	**0.040**
*P* × *W*	$\boldsymbol{\lt0.001}$	$\boldsymbol{\lt0.001} $
*M* × *P* × *W*	0.54	0.83

There were ten repetitions of the ten stimuli, producing a total of 100 stimulation trials per subject inside the scanner and 100 trials outside the scanner. The stimuli were delivered every 8–12 s. The stimuli were drawn from the 100-stimulus set randomly without replacement. This way, stimulus order could not affect the results.

### 2.8. Responses and their assessment

Subjects were instructed to report a percept with a verbal command of any combination of $\{$Pain, Vibration, Tap$\}$, and their intensity (1: low, 2: medium, 3: high). Following each stimulus, the experimenter was prompted to entered the reported sensation (or lack thereof) and its intensity into a command line of the same Matlab program that scheduled the stimuli. The experimenter was blinded to the stimuli. Following the experiment, for each stimulus type, the response magnitude was computed as the proportion of trials in which subjects’ registered a response, weighted by the reported intensity. The principal results were the same regardless of whether the percepts were weighted by their intensity or were considered binary (see section 3). Vibration and tap responses were grouped together as tactile.

### 2.9. Acoustic continuum

The acoustic impedance of water and skin, including soft tissues, are closely matched (1.48 MRayl compared to 1.68 MRayl Kuhn *et al* 2008). This way, about 99.6% of the energy, $1-R^2 = 1-\left(\frac{1.68-1.48}{1.68+1.48}\right)^2$, was delivered into the finger. The water-finger interface is therefore essentially acoustically transparent and can be considered as a continuum from the perspective of ultrasound.

### 2.10. Stimulus safety

The ultrasonic stimuli used in this study were safely below the FDA 510(k) Track 3 recommendations (FDA 2023). In particular, the highest peak pressure used in the study, 0.7 MPa, corresponds to peak intensity of 15.3 W cm^−2^, which is well below the FDA recommendation of $I_\textrm{SPPA} = 190$ W cm^−2^ (table 2). In addition, the time-average spatial peak intensity was $I_\textrm{SPTA} = 150$ mW cm^−2^, also below the FDA recommendation of $I_\textrm{SPTA} = 720$ mW cm^−2^. The computation of the charge density (table 2) assumed brain conductivity of 0.26 S m^−1^ (Koessler *et al* 2017). Thus, stimuli of much higher levels could be used, from both the ultrasound safety and electrical stimulation safety perspectives. The 0.7 MPa maximum allowed all sensations to be tolerated by the subjects. The thumb function was normal following the experiments and its sensation remained unaffected in all subjects.

**Table 2.** Compliance with safety indices. The study used nine distinct stimuli: three levels of pressure and three distinct waveforms, one continuous (100% duty) and two pulsed (both 50% duty). All stimuli were 200 ms in duration and were delivered every 10 s on average. The study followed the FDA 510(k) Track 3 recommendations (FDA 2023): peak intensity $I_\textrm{SPPA}$ and time-average intensity $I_\textrm{SPTA}$. *E* is the induced peak Lstim intensity in a 7 T magnetic field. The computation of the charge density assumes brain conductivity of 0.26 S m^−1^ (Koessler *et al* 2017). Electrical stimulation should ideally not exceed charge density of 30 *µ*C cm^−2^ (Cogan *et al* 2016). All stimuli are within the recommended safety levels.

Pressure (MPa)	*E* (V m^−1^)	Waveform	On (ms)	Off (ms)	*I*_SPPA_ (W cm^−2^)	*I*_SPTA_ (W cm^−2^)	Charge density (*µ*C cm^−2^)
0.35	1.53	Pulsed	100	9900	3.8	0.0	1.4
0.53	2.30	Pulsed	100	9900	8.6	0.0	2.1
0.70	3.06	Pulsed	100	9900	15.3	0.1	2.8
0.35	1.53	Continuous	200	9800	3.8	0.0	2.8
0.53	2.30	Continuous	200	9800	8.6	0.1	4.2
0.70	3.06	Continuous	200	9800	15.3	0.2	5.6
		Safety guidelines			190	0.72	30

### 2.11. Non-human primate brain stimulation

Two adult male rhesus non-human primates ($Macaca~mulatta$) participated in the brain stimulation. All procedures complied with an approved Institutional Animal Care and Use Committee protocol of the University of Utah. The ultrasound was delivered using a 256-element, MRI-compatible phased array detailed in a dedicated publication (Webb *et al* 2022). Briefly, the transducer array is inserted into a frame that is mounted into four titanium pins attached to the skull. This mounting system ensures reproducible targeting (Webb *et al* 2022, 2023). Coupling to the head is mediated using a cryogel. The coupling quality is validated prior to each session using an ultrasound imaging sequence (Webb *et al* 2022). The animals were positioned inside MRI in a standard sphinx position. The animals were anesthetized with isoflurane (1.0–1.25% + 1–2 l min^−1^ medical grade O2). The ultrasound was delivered into two deep brain targets, the left and right lateral geniculate nucleus (LGN). Targeting of the LGN was validated using MRI thermometry (Webb *et al* 2022, 2023). Ultrasound stimuli (100 ms duration, 480 kHz carrier frequency, 2 MPa amplitude *in situ* Webb *et al* 2022) were applied to each LGN every 4 s to in a strictly alternating manner (left LGN, right LGN, etc., every 4 s). The stimuli were either continuous or pulsed at 200 Hz pulse repetition frequency, 50% duty cycle. Data were collected for the animals fully positioned inside a 3 T MRI (Siemens TRIO and VIDA) or translated such that the head lied 2 m outside of the entry plane to the bore. The magnetic field at that distance comprised about 20 mT. Each animal underwent two stimulation sessions. In monkey 1, the order of the pulsed stimulation was inside then outside for both sessions. For the CW sonications, the order in the first session was inside then outside, and the order was reversed in the second session. In animal 2, the order of the pulsed stimulation was inside then outside for the first session and reversed for the second session. In this animal, only one CW session was recorded and the order of that session was outside then inside. There was at least a 2 min interval between the inside and outside stimulation. Each session delivered 40 stimuli in total. This number was determined to provide sufficient statistical power while not imparting potentially detrimental effects on the stimulated tissue. We recorded a total of seven sessions (monkey 1: two sessions pulsed stimulation, two sessions continuous stimulation; monkey 2: two sessions pulsed stimulation, one session continuous stimulation), each contrasting the presence and absence of the magnetic field. The ultrasound pressure amplitude of 2.0 MPa, corresponding to $I_\textrm{SPPA} = 129.0$ W cm^−2^ lied within the $I_\textrm{SPPA} = 190$ W cm^−2^ recommendation of the 510k guidelines (FDA 2023).

The recordings and the quantification of gamma activity were analogous to a previous study (Webb *et al* 2023). The activity was assessed over 400 ms windows, overlapping every 100 ms. The gamma activity was normalized by the average gamma activity within a 1 s window preceding each stimulus, which provided a baseline for the assessment of the ultrasound and Lstim-evoked changes. Evoked activity was averaged over both posterior posts and over the left and right LGN stimuli.

## 3. Results

The equation that governs the generation of electric field from ultrasonic and magnetic fields, $E\,=\,\frac{PB}{Z}$, predicts that the generated electric field intensity should scale with the ultrasound pressure at the rate of $\frac{B}{Z}$ (figure 1(B), solid). In line with this prediction, we measured (figure 1(B), dashed) a significant increase of the evoked electric intensity with ultrasound pressure (*p* = 0.037, F-test of linear regression). The slope of the measured field, 5.5 V m^−1^ MPa^−1^, was in good agreement with that computed using the Lorentz equation (4.4 V m^−1^ MPa^−1^) for the applicable magnetic field strength (7 T). Therefore, perpendicular applications of magnetic and ultrasonic fields indeed generate electric fields as predicted by the Lorentz equation, thus providing a direct validation of the concept (Norton 2003, Yuan *et al* 2016).

Figure 1 demonstrates that standard hardware can produce Lfield intensities that are relevant to biological applications. A 0.5 MPa stimulus, well within the FDA 510(k) safety indices (FDA 2023), evokes inside a 7 T field peak electric intensity of 2.81 V m^−1^ (figure 1(B)). Such field strength can appreciably modulate neural activity (Liu *et al* 2018). Fields as low 0.3 V m^−1^ have been shown to modulate neuronal spiking (Francis *et al* 2003). The clinically-relevant transcranial electrical stimulation produces about 0.28 V m^−1^ (95th percentile) in the human brain (Huang *et al* 2017) for the generally accepted maximum current of 2 mA.

We applied the same hardware to test whether Lstim can modulate bioelectric signaling. Specifically, we focused ultrasound from a distance of 52 mm onto a target that features intact nerves and receptors—the human thumb (figure 2(B)). Focused ultrasonic stimuli (figures 2(C) and (D)) were delivered into the target every 8–12 s. Subjects (*n* = 18) were asked to report any nociceptive or tactile sensation. A nociceptive sensation results from activation of free nerve endings in the skin (Dubin *et al* 2010) and thus constitutes a metric of neural activation.

**Figure 3.**
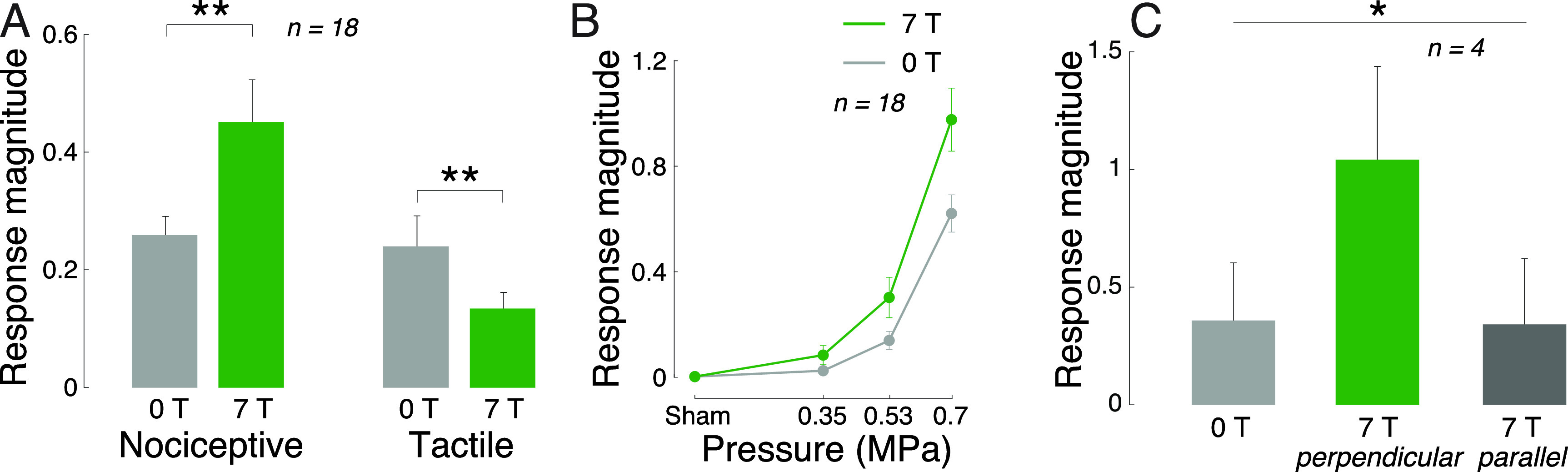
Remote targeted modulation of the peripheral nervous system. (A) Lstim modulates human peripheral nervous system. Mean ± s.e.m. response magnitude (see section 2) for ultrasound alone (0 T) and ultrasound combined with magnetic field (7 T), separately for nociceptive (left) and tactile (right) responses. Data were pooled over all stimuli tested. The double stars indicate effects significant at *p* < 0.01. (B) Lstim-evoked nociceptive responses increase with ultrasound pressure. Mean ± s.e.m. magnitude of nociceptive responses as a function of ultrasound pressure at target and the presence (green) and absence (gray) of magnetic field. Data were pooled over all stimuli. (C) Lstim activates nerves in an orientation-specific manner. Mean ± s.e.m. magnitude of nociceptive responses as a function of the orientation of the induced electric field with respect to the subjects’ nerves. The neuromodulatory effects are maximized when the nerves are aligned with the induced electric field (green). Data were pooled over all stimuli. The star indicates that the modulation by the magnetic field and its orientation was significant (*p* < 0.05).

We found that the magnetic field substantially enhanced the magnitude of nociceptive responses (figure 3(A)). Across all pressure levels and waveforms, Lstim increased the magnitude of nociceptive responses by 74%. In contrast to nociceptive responses, tactile responses were suppressed (figure 3(A)); there was a double dissociation of the effects with respect to magnetic field and the sensation kind (two-way ANOVA, magnetic field × sensation interaction, *p* < 0.001; $F(1,644) = 13.20$). The effect was similar when subjects’ responses were not scaled by their intensity (*p* < 0.001; $F(1,644) = 13.93$). Pairwise post-hoc tests showed that the increase in the nociceptive responses (*p* = 0.0059; $t(17) = 3.14$, paired two-sided t-test) and the decrease in tactile responses (*p* = 0.0033; $t(17) = -3.41$) were significant. These effects were also similar when the responses were not scaled by their intensity (*p* = 0.0037; $t(17) = 3.36$ and *p* = 0.0029; $t(17) = -3.47$, respectively).

We next specifically analyzed the nociceptive responses, which reflect an activation of nerves or nerve endings (Dubin *et al* 2010). Figure 3(B) shows the dependence of all stimuli on the presence or absence of magnetic field, separately for each ultrasound pressure. The figure confirms the findings of figure 3(A) that the magnetic field amplifies the nociceptive responses. We assessed the effects using a full, three-way ANOVA model with factors magnetic field, ultrasound pressure, stimulus waveform, and all possible interactions (table 1). The effect of magnetic field was significant also in this omnibus analysis (*p* < 0.001, $F(1,408) = 18.55$).

Lstim produces focused electric fields at ultrasound targets according to $E = \frac{P B}{Z}$. In this equation, the effect increases with the ultrasound pressure *P*. Therefore, the higher the ultrasound pressure, the stronger the induced electric fields, and the stronger the nociceptive responses we should observe, in addition to any neuromodulatory effects of ultrasound alone. In line with this expectation, we found a significant interaction between the magnetic field and ultrasound pressure (figure 3(B)); *p* = 0.0012, $F(3,408) = 5.41$).

We summarize the effects of all factors and interactions in table 1. With respect to nerve activation, as assessed by the nociceptive responses, there was a significant interaction between magnetic field and the stimulus waveform (*p* = 0.043, $F(2,408) = 3.16$). The contrast between Lstim and ultrasound only was higher when the ultrasound was pulsed. Specifically, averaged across all pressures, the response frequency ratio (7 T versus 0 T) for the continuous waveform was 1.61, compared to 1.85 and 3.85 for the pulsed 500 Hz and 10 kHz waveforms, respectively.

If the reported effects are indeed due to the induction of localized electric field, as governed by the Lorentz electromotive force equation, they should depend on the orientation of the nerves with respect to the electric field. Specifically, electric fields can effectively stimulate nerves if their gradients point along nerves, as opposed to across (Rattay 1999). To test this, four subjects were asked to place their thumb on the aperture (1) perpendicularly to the magnetic field (the current default) and (2) in parallel with the magnetic field. We found (figure 3(C)) that these conditions significantly modulated the responses (*p* = 0.041, $F(2,33) = 3.50$). As expected, the effect was specific to the perpendicular geometry; there was no effect for the parallel geometry (*p* = 0.88, $t(3) = 0.17$, paired two-sided t-test).

The stimuli used in this study were designed to comply with the applicable safety indices (table 2). Specifically, all ultrasonic stimuli were safely within the FDA 510(k) indices (FDA 2023). Furthermore, the induced electric fields lied safely below the recommended charge density (Cogan *et al* 2016) of 30 *µ*C cm^−2^ (table 2). There were no detrimental acute or long-term effects reported by the subjects.

**Figure 4.**
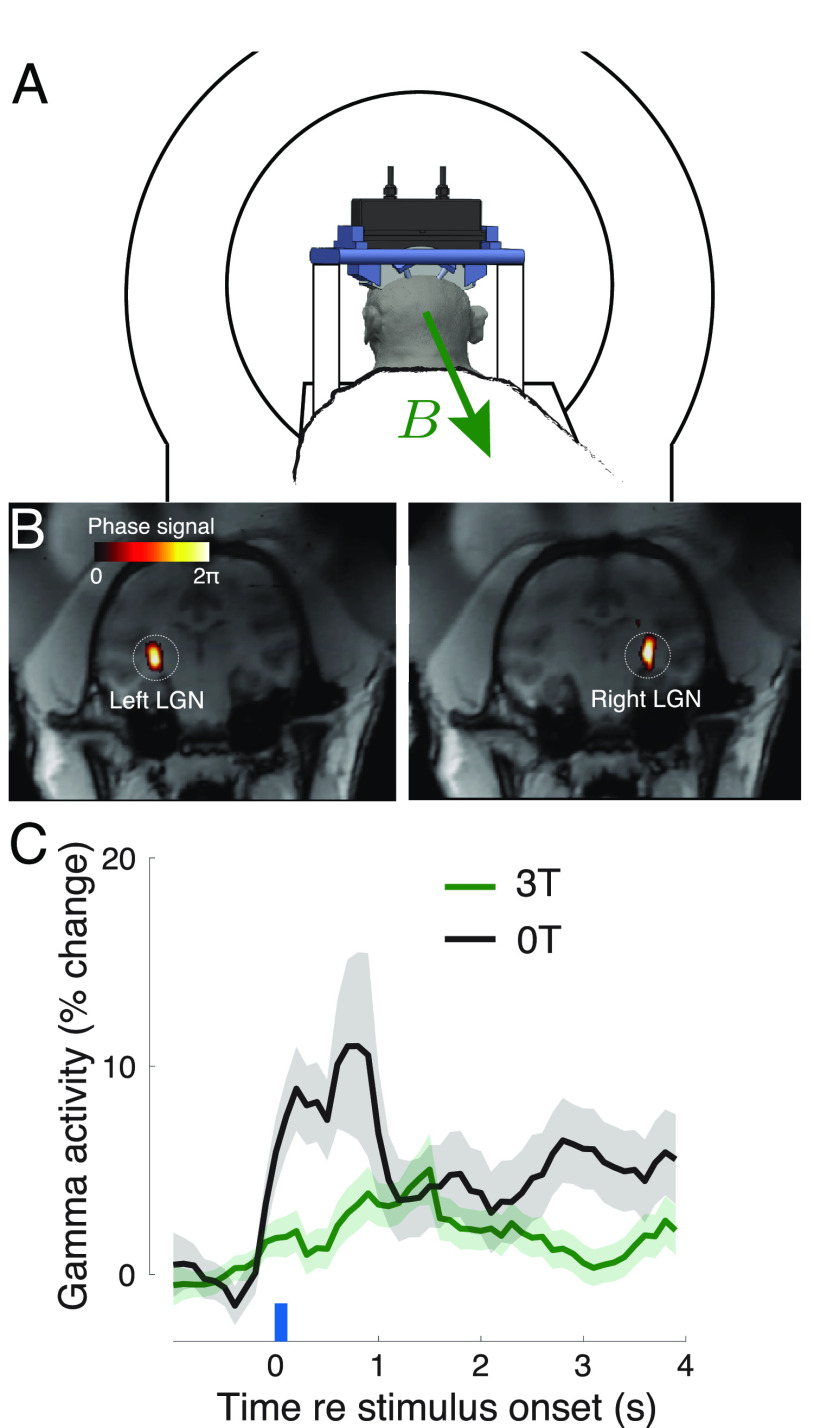
Remote targeted modulation of the brain. (A) A 256-element, MRI-compatible phased array (Webb *et al* 2022, 2023) is inserted into a frame that is mounted into four titanium posts attached to the skull of two non-human primates. Each animal was positioned in a standard sphinx position. In this position, the magnetic field of the scanner (green arrow) points toward the reader. Since the ultrasound is delivered from the top, the induced Lstim field points along the animal’s left-right axis. (B) Example validation of the LGN targeting using MRI thermometry. The images represent the selective targeting of the left and right LGN (Webb *et al* 2022, 2023) (C) Mean ± s.e.m. high gamma activity recorded from the two posterior pins in response to 100 ms stimuli (480 kHz carrier frequency, 2 MPa amplitude) applied every 4 s to each LGN in a strictly alternating manner. The stimuli were either continuous or pulsed at 200 Hz pulse repetition frequency. Since there was no statistically significant difference, we pooled the data across these two conditions. Data are shown separately for the animals positioned inside the MRI (green) and 2 m outside of the MRI bore (black). The responses are aligned to the offset of each ultrasound stimulus (blue bar) and contain data of seven sessions recorded in the two monkeys.

We finally evaluated the effects of Lstim on deep brain regions of non-human primates. Specifically, we targeted the lateral geniculate nuclei (LGN), deep brain regions that pass visual information to visual cortex (figure 4(B)). Our previous study (Webb *et al* 2023) showed that ultrasonic neuromodulation of the LGN increases gamma activity over visual cortex. We therefore used the same apparatus and recordings to assess the effects of Lstim. The stimulation was delivered inside and outside of a static field of a 3 T MRI magnet every 4 s. The ultrasonic stimuli were 2 MPa in amplitude, 100 ms in duration, and were either continuous or pulsed at 200 Hz pulse repetition frequency. Replicating our previous findings (Webb *et al* 2023), we found a robust increase in gamma activity over visual cortex following the ultrasonic stimulation (figure 4(C); black). Crucially, the presence of the strong magnetic field had a profound influence on the induced gamma activity (figure 4(C); green). In particular, the presence of the magnetic field dampened the gamma response, and led to a much more gradual increase following the stimulus onset. We assessed these effects using a two-way ANOVA, with factors magnetic field and stimulus type (continuous or pulsed). We measured the gamma activity in the time window immediately following the ultrasound offset (100 ms) up until the end of each trial (time 4 s). There was a significant effect of magnetic field ($F(1,981) = 5.64$, *p* = 0.018). Stimulus type or the interaction of the two factors were non-significant ($F(1,981) = 1.27$, *p* = 0.26 and $F(1,981) = 1.06$, *p* = 0.30, respectively). In the time window considered, there was an average increase of gamma by 6.1% and 5.4% in monkeys 1 and 2 at 0 T, compared with 2.0% and 2.5% at 3 T. No detrimental effects were observed during or after the stimulation. The animals showed normal behavior following the procedures.

## 4. Discussion

This study shows that the combination of magnetic and focused ultrasonic fields generates localized electric fields remotely and noninvasively. The resulting stimulation, Lstim, produces notable neuromodulatory effects using standard hardware in the peripheral nervous systems of humans and the central nervous system of non-human primates. The method can therefore be deployed for electrode-free modulations of neural and other processes that rest on electrical signaling.

Lstim provides three major advantages compared with traditional, electrode-based stimulation. The first, key advantage is that Lstim does not require the insertion of electrodes into a target to produce localized electric fields. The localization is achieved through the focusing of ultrasound. For high ultrasound frequencies, the stimulation focus can be as tight as a few dozens of micrometers (Menz *et al* 2013). Second, Lfield produces much sharper gradients and thus has a much higher potential for triggering bioeffects compared with electric fields generated with a pair of electrodes. Specifically, the Lorentz equation $E(x,y,z) = P(x,y,z)\frac{B}{Z}$ shows that the spatial distribution of the electric intensity $E(x,y,z)$ follows the distribution of the ultrasonic pressure wave $P(x,y,z)$. Consequently, the propagating sinusoidal ultrasound pressure wave generates an *E* gradient with the peak positive and peak negative *E* values spaced by $\frac{\lambda}{2}$. At 258 kHz, this amounts to ${\approx}2.9$ mm. In comparison, traditional electromagnetic fields, due to their much higher speed of propagation, have a $\frac{\lambda}{2} \approx 231$ m for the same frequency. Thus, thanks to the ultrasonic component, Lstim generates in the target electric field gradients that are five orders of magnitude stronger compared to traditional electromagnetic fields. This difference is critical with respect to neurostimulation as neurostimulation effects are known to scale with an activating function $f \propto \frac{\mathrm dE}{\mathrm dx}$, where $\frac{\mathrm dE}{\mathrm dx}$ is the gradient of the electric fields along the excitable structure (Rattay 1999). The Lfield gradients can be controlled by specific frequencies and waveforms of the ultrasonic pressure wave. And third, Lstim circumvents the barriers associated with biological membranes. Membranes are transparent to Lstim. This is because membranes are transparent to magnetic and ultrasonic fields (Cobbold 2006). Lstim’s independence of biological membranes may open a new set of applications that modulate intracellular processes remotely and more effectively than previously possible.

We detected notable effects on peripheral nerves in humans at ultrasound pressures approximately three times lower than those allowable by current FDA 510(k) guidelines (0.7 MPa compared with 2.4 MPa or $\lt190$ W cm^−2^ in soft tissues FDA 2023). At 2.4 MPa, still considered safe, the effects of Lstim would be more than three times stronger than those reported here. Moreover, for the relatively low frequencies such as those used here, ultrasound amplitudes higher than 2.4 MPa may be applied in brief pulses without a risk of harmful heating (Downs *et al* 2018). If even stronger effects are needed for certain applications, the stimulation could be performed in stronger magnetic fields. Magnetic fields over 30 T are readily available (Battesti *et al* 2018).

We found that Lstim increased nociceptive responses and decreased tactile responses. The preferential engagement of nociceptive fibers is likely due to the sharp, mm-level gradients induced by Lstim. The gradients are likely to preferentially engage nerve structures with geometries on that order (i.e. nociceptive fibers), while the effects may average out over geometries that exceed the wavelength (i.e. tactile receptors and fibers). Moreover, this double dissociation suggests that Lfields modulated the electrical signals generated by skin receptors in response to ultrasound (Riis and Kubanek 2021). This modulation should be studied in detail in the future as it may have important applications for blockage of aberrant signaling, such as that involved in pain.

We delivered into the deep brain targets of non-human primates pressures of 2.0 MPa to induce reliable changes in gamma activity recorded over visual cortex. We found that that the presence of 3 T magnetic field substantially dampened and slowed the gamma responses to ultrasound. The effect was present for at least 4 s, the duration of the inter-stimulus interval. High-frequency continuous electrical waveforms have been used for neural inhibition or conduction block (Kilgore and Bhadra 2014, Grossman *et al* 2017). The relatively high carrier frequency of the ultrasound produces high-frequency waveforms, and thus the effect is consistent with that literature. It is possible that the relatively high pressure amplitude of 2.0 MPa further amplified this effect. It is also likely that peripheral nerves and LGN neurons respond to this new mode of stimulation, which induces sharp gradients, in fundamentally distinct ways. These findings warrant the study of this phenomenon systematically for each specific neural structure.

We hypothesized that continuous stimuli, which consist of a high-frequency carrier, would suppress neural activity (Kilgore and Bhadra 2014) while pulsed stimuli would produce an ‘onset response’—transient increases in neural activity following the onset of a high-frequency stimulus (Grossman *et al* 2017). Yet, both the human peripheral and the monkey central nervous systems did not distinguish between the stimulus types. It is possible that the induction of a pronounced neural excitation with Lstim will require much higher pulse repetition frequencies than those considered here.

The noninvasive and targeted nature of Lstim provides a new means for systematic modulation of specific neural targets in each individual, with the potential to realize the promise of precision medicine. Existing arrays of transducers focus ultrasound programmatically into neural targets that can be as small as a few dozens of micrometers when applied in soft tissues (Menz *et al* 2013), and about 3 mm in diameter when applied through the human skull (Ghanouni *et al* 2015). Coupled with the microsecond-level temporal resolution of ultrasound, Lstim can activate multiple circuits in sequence or in concert. Together, the high spatiotemporal resolution of the method provides the means to modulate specific neural targets systematically. For instance, clinical teams could use Lstim to identify the neural circuits that are involved in chronic pain of a particular individual. In addition, the ability to systematically manipulate specific brain circuits has the potential to transform our understanding of basic brain function. Because Lstim uses energy levels well within recommended guidelines (table 2), such systematic applications are expected to be safe. Indeed, ultrasound has been applied to the human brain inside strong magnetic fields, up to 7 T, and no detrimental effects were reported (Lee *et al* 2016, Ai *et al* 2018).

For static magnetic fields, Lstim produces stimulation of the same frequency as that of the applied ultrasound. The defined ultrasound frequency make the approach immune to potential influence of external sources, unless they operate at the same frequency as that of the ultrasound.

Lstim may require two additional innovations for practical deployment in clinical and research settings. First, Lstim requires a strong magnetic field and so operation inside an MRI machine. This increases the cost and complicates the logistics of deployment. This issue could be addressed by producing the magnetic field using custom coils. Since no gradients and imaging are required, such systems could be produced much more affordably than an MRI scanner. And second, the human skull attenuates ultrasound strongly and shows high subject-to-subject variability in the attenuation Riis *et al* (2022). Approaches that address this issue in a noninvasive way are being developed (Riis *et al* 2022).

Lstim induces electric fields without the need for inserting electrodes into the target, thus preserving its integrity and sterility. This may lead to applications beyond neuromodulation, such as remote stimulation of tissue or cell cultures (Hu *et al* 2019), food processing, or the catalysis of certain chemical reactions.

In summary, this study shows that remote application of magnetic and ultrasonic fields produces electrical stimulation that is both effective and safe. The high spatiotemporal resolution of the stimulation, enabled by existing ultrasound phased array technology, provides a new means to modulate biological processes flexibly and systematically. This systematic tool is expected to accelerate basic biological research and enable noninvasive modulations of spatially specific biological processes, including those in the nervous system.

## Data Availability

The data that support the findings of this study will be openly available following an embargo at the following URL/DOI: https://nda.nih.gov/. Data will be available from 18 August 2023.
